# Complete Reversible Refolding of a G-Protein Coupled Receptor on a Solid Support

**DOI:** 10.1371/journal.pone.0151582

**Published:** 2016-03-16

**Authors:** Natalie Di Bartolo, Emma L. R. Compton, Tony Warne, Patricia C. Edwards, Christopher G. Tate, Gebhard F. X. Schertler, Paula J. Booth

**Affiliations:** 1 School of Biochemistry, University of Bristol, Bristol, United Kingdom; 2 Organisational and Staff Development Unit, University of Strathclyde, Glasgow, United Kingdom; 3 MRC Laboratory of Molecular Biology, Cambridge Biomedical Campus, Cambridge, United Kingdom; 4 Laboratory of Biomolecular Research, Paul Scherrer Institute, Villigen, Switzerland; Universidad de Granada, SPAIN

## Abstract

The factors defining the correct folding and stability of integral membrane proteins are poorly understood. Folding of only a few select membrane proteins has been scrutinised, leaving considerable deficiencies in knowledge for large protein families, such as G protein coupled receptors (GPCRs). Complete reversible folding, which is problematic for any membrane protein, has eluded this dominant receptor family. Moreover, attempts to recover receptors from denatured states are inefficient, yielding at best 40–70% functional protein. We present a method for the reversible unfolding of an archetypal family member, the β_1_-adrenergic receptor, and attain 100% recovery of the folded, functional state, in terms of ligand binding, compared to receptor which has not been subject to any unfolding and retains its original, folded structure. We exploit refolding on a solid support, which could avoid unwanted interactions and aggregation that occur in bulk solution. We determine the changes in structure and function upon unfolding and refolding. Additionally, we employ a method that is relatively new to membrane protein folding; pulse proteolysis. Complete refolding of β_1_-adrenergic receptor occurs in n-decyl-β-D-maltoside (DM) micelles from a urea-denatured state, as shown by regain of its original helical structure, ligand binding and protein fluorescence. The successful refolding strategy on a solid support offers a defined method for the controlled refolding and recovery of functional GPCRs and other membrane proteins that suffer from instability and irreversible denaturation once isolated from their native membranes.

## Introduction

A central feature of biological activity is the correct folding of proteins into their functional states. Integral membrane proteins have been largely overlooked in the extensive investigations levelled at understanding this folding process. Biophysical studies *in vitro* are currently the method of choice for elucidating molecular level mechanistic detail [1–6] and these are being complemented by studies using cellular membrane extracts to capture the additional cellular machineries involved in correct membrane protein folding *in vivo* [7–10]. One of the largest knowledge gaps lies with biophysical studies of transmembrane helical proteins and there is a dearth of information on many large membrane protein families. A case in point are GPCRs. These constitute the largest family of membrane receptors, with over 800 human GPCRs identified, and are the focus of considerable research and industrial interest [11, 12]. The primary function of GPCRs is to respond to extracellular stimuli and activate an array of intracellular signalling pathways through their interaction with numerous G protein subtypes as well as through G-protein independent pathways, often in a ligand-specific manner [13, 14]. The lack of *in vitro* folding information on this particular protein class has significant ramifications. It impacts directly upon achieving and maintaining a correctly folded, functional structure for the plethora of biophysical, biochemical and structural work aimed at ascertaining functional mechanisms for GPCRs. Obtaining sufficient quantities of properly folded GPCRs for *in vitro* structural and functional studies has proven tremendously difficult, not least due to the poor long-term stability of the receptors in detergents and associated loss of structure and function. These practical issues, together with the inherent importance of understanding folding, have highlighted the significance of investigating GCPR refolding and stability. One of the most promising advances in this area has been the use of columns as solid supports for recovering functional GPCR from inclusion bodies, using SDS to solubilise the inclusion bodies generally with amphipols or bicelles to recover folded protein [15–19]. The most successful cases result in ~40–70% recovery of functional protein. Solubilisation of inclusion bodies in urea gives much lower yields of 25%. The extent of denaturation of the SDS or urea-solubilised state has not been assessed in detail—these denaturants could in fact be primarily solubilising protein that is already largely folded from the inclusion bodies, so little or no refolding is required. Nor have there been any reports on the controlled, reversible unfolding and refolding a receptor. Reversible unfolding has however, been demonstrated for several other α-helical membrane proteins from different protein classes including diacylglycerol kinase (DGK) [20], the potassium channel, KscA [21, 22], the galactose transporter, GalP [3] and the small multidrug resistance transporter, EmrE [1].

Here, we present a systematic folding study of the thermostabilised turkey β_1_-adrenergic receptor (β_1_AR-m23) [23, 24], using urea and SDS denaturants. This mutant offers an exciting opportunity for folding studies as the increased stability makes refolding a viable prospect. We compare refolding in detergent micelles composed of DM in bulk solution with a solid support. The latter approach gives unprecedented reversible refolding of the GPCR, from a urea-denatured state, with a 100% yield. Although interactions with other lipids or proteins in native membranes may be important for the functional folded state of GPCRs *in vivo*, here we study the receptor in isolation and address function with respect to ligand binding only. It is vital to attain complete recovery of such function as membrane proteins are notorious for perturbations of ligand binding constants and reduced stability when solubilised in detergents, even without any refolding.

## Materials and Methods

### Materials

DM was purchased from Anatrace Inc. (Maumee, OH, USA). DMPC was from Avanti Polar Lipids Inc (Alabaster, Al). CHAPS was from Calbiochem. [^3^H] (-) dihydroalprenolol (DHA) was from Amersham. Ni-NTA agarose beads were from Qiagen. All other chemicals were of the highest grade and purchased from Sigma-Aldrich, unless stated otherwise.

### Protein expression and purification

Purified β_1_AR-m23 was kindly provided by the laboratory of Chris Tate (MRC, Laboratory of Molecular Biology). The β_1_AR-m23 construct is truncated at both the N- (residues 3–32) and C-terminus (after residue Leu367) and contains a C-terminal tag of six histidine residues for purification. A segment, comprising residues 244–271, is also deleted. The construct contains eight point mutations: C116L improves expression; C358A at the C-terminus of Helix 8 removes a palmitoylation site; R68S, M90V, Y227A, A282L, F327A and F338A improve thermal stability. The receptor was expressed using the baculovirus system in High 5 cells. Membrane preparation, solubilisation, immobisilied metal ion affinity chromatography (IMAC) and alprenolol ligand-affinity chromatography were all performed as previously described [23]. The receptor was purified in buffer containing 20 mM Tris pH 7.8, 350 mM NaCl, 0.2 mM EDTA, 100 nM alprenolol and 0.1% decylmaltoside (DM).

### Urea unfolding

Fluorescence and CD unfolding experiments were carried out at room temperature at a final β_1_AR-m23 concentration of 0.45 μM (0.0162 mg/ml) and 4.5 μM (0.162 mg/ml). For urea unfolding experiments in DM, β_1_AR-m23 was diluted into buffer containing 25 mM Tris pH 7.5, 150 mM NaCl, 0.1 mM EDTA, 0.5% DM and varying concentrations of urea (0–9 M) and incubated for 30 min with gentle mixing before spectral analysis was carried out. A higher concentration of DM was required in unfolding experiments carried out in urea, than SDS, due to the effects of urea on increasing the cmc of the detergent. Where possible averaged results are from independent experiments performed using different samples but the same protein preparation. However, in those experiments requiring large amounts of sample, such as CD, averaged results are sometimes from independent experiments using samples from different protein preparations. Variations in the CD signal and fluorescence were observed between different preparations of protein, most likely due to errors in determining protein concentration.

### Refolding from a urea-denatured state

β_1_AR-m23 was refolded by immobilisation on a Ni^2+^ affinity resin. β_1_AR-m23 was diluted to approximately 4.5 μM in 2 ml of DM buffer (25 mM Tris pH 7.5, 150 mM NaCl and 0.5% DM) and incubated with 1 ml Ni^2+^-NTA agarose for 1.5 h at 4°C. Ni^2+^-NTA agarose beads were recovered by centrifugation and washed with buffer containing 8 M urea for denaturation of the protein. The beads were again recovered and refolding initiated by washes with 3 x 2 ml of DM buffer. Refolded β_1_AR-m23 was eluted in DM buffer containing 300 mM imidazole. For folded control experiments, the DM buffer was unchanged. For unfolded control experiments, refolding was not initiated and β_1_AR-m23 was eluted in DM buffer containing 8 M urea and 300 mM imidazole. The imidazole was removed from all samples using a PD MiniTrap G-25 column (GE Healthcare) and protein concentration determined using the Markwell-Lowry assay [25]. Refolding of urea-unfolded β_1_AR-m23 was also attempted by rapid dilution into DM. β_1_AR-m23 was unfolded by dilution into 25 mM Tris pH 7.5, 150 mM NaCl, 0.5% DM or and 8 M urea to a final concentration of 4.5 μM. Following incubation at room temperature for 5 min refolding was initiated by a tenfold dilution into buffer without denaturant and incubated for a further 30 min. The protein signal was too low to be accurately detected following refolding from a lower protein concentration of 0.45 μM, this was especially true when refolding was carried out using the dilution method where the protein is diluted 10-fold into a renaturing buffer.

### Fluorescence spectroscopy

Intrinsic protein fluorescence emission spectra were recorded on a Fluoromax-2 (Jobin Yvon) at room temperature and in a 10 mm pathlength cell. Samples were excited at 280 nm and emission spectra collected between 295–450 nm with 1 nm excitation and 5 nm emission bandwidths. Unfolding curves were generated by plotting the red-shift in the fluorescence emission maximum against denaturant concentration.

### Circular dichroism spectroscopy

CD spectra were measured at room temperature between 200–260 nm in 1 nm intervals with a 1 s integration time and a band width of 1 nm. Cells with pathlengths of 5 and 0.5 mm were used for protein concentrations of 0.45 and 4.5 μM, respectively. Data was analysed using CDtools software [26] and unfolding curves were generated by plotting the reduction in helicity, calculated from changes in the CD signal at the 222 nm band which is characteristic of α-helical proteins, against denaturant concentration. The 222 nm band was used to monitor folding due to the absorbance of urea at low wavelengths.

### Radioligand binding assays

Saturation binding assays were carried out with [^3^H](-) dihydroalpenolol essentially as previously described [27]. Briefly, assays were performed in 25 mM Tris pH 7.5, 150 mM NaCl and 0.5% DM with 60 nM [^3^H](-) DHA in a final volume of 120 μl which were incubated on ice for 1 h. Bound and free radioligand were then separated by centrifugal gel filtration using columns packed with 4.4 ml sephadex G-25M (GE Healthcare) pre-equilibrated with DM buffer as above. Tritiated antagonist was determined by liquid scintillation counting. Non-specific binding was determined in the presence of 10 μM s-propranolol to folded and unfolded protein samples and also incubated on ice for 1 h. In urea unfolding experiments measuring the ligand binding activity of refolded β_1_AR-m23, binding assays were performed after the refolding procedure on the Ni^2+^ affinity resin (see the Materials and Methods above). The activity of the refolded receptor was compared to that determined of the originally purified receptor that had not been denatured by urea. In these folded control experiments the receptor was bound to the column but not treated with urea and instead the DM-buffer remained unchanged. Unfolded control experiments were also performed in which β_1_AR-m23 was eluted in 8 M urea. Following elution from the column and determination of protein concentration by the Markwell-Lowry assay, the receptor was diluted to 0.1 μM in the appropriate DM-containing buffer before addition of 5 μl (~18 ng) to each radioligand binding assay (120 μl total reaction volume) giving a final receptor concentration of 4.2 nM (with the radioligand being 60 nM).

The ability of β_1_AR-m23 to bind radioligand following immobilisation and urea unfolding on a Ni^2+^ resin was also measured. 4.5 μM β_1_AR-m23 was bound to Ni^2+^-NTA agarose in DM as previously described in the Materials and Methods. Ni^2+^-NTA agarose beads were recovered by centrifugation and unfolding of β_1_AR-m23 initiated by solvent exchange to buffer containing 8 M urea. Following 1 min incubation, beads were recovered and resuspended in buffer containing 8 M urea and 0.6 μM [^3^H] (-) DHA. Following a further minute incubation, beads were again recovered and a wash step in 8 M urea performed to remove any unbound radioligand. At the end of the experiment, the beads were recovered, resuspended in 8 M urea and the radioactivity associated with the beads, and therefore bound to β_1_AR-m23, measured. It should be noted that the total unfolding time with 8 M urea was 5 minutes. Control experiments were also carried out with no protein and with β_1_AR-m23 but under folding conditions by replacing buffer containing 8 M urea with buffer in which urea had been omitted.

### Pulse proteolysis

The use of pulse proteolysis to follow β_1_AR-m23 unfolding was initially tested in bulk solution using a 1-min pulse as follows; β_1_AR-m23 was diluted into buffer containing 25 mM Tris pH 7.5, 150 mM NaCl, 0.5% DM, 10 mM CaCl_2_ and urea (2–8 M) at a final concentration of 0.45 μM and allowed to equilibrate for 30 min at room temperature. Proteolysis was initiated by adding a 50x stock solution of thermolysin in 2.5 M NaCl and 10 mM CaCl_2_ to a final concentration of 0.2 mg/ml. After 1 min incubation, the reactions were quenched by the addition of 13.3 μM phosphoramidon and vortexed. Quenched reactions were analysed on 12% (w/v) SDS-PAGE stained with SYPRO Red fluorescent dye (Molecular Probes). Band intensities were quantified with the image analysis software AlphaErase FC. Pulse proteolysis was also used to monitor β_1_AR-m23 unfolding on a Ni^2+^-NTA column and compared to that in bulk solution. In these experiments, 0.45 or 4.5 μM β_1_AR-m23 in either bulk solution or bound to Ni^2+^-NTA agarose beads was incubated in buffer as above containing 3 or 8 M urea for 2 or 27 min (for a 5 or 30 min total unfolding time). Proteolysis was initiated by the addition of thermolysin and quenched by the addition of phosphoramidon, as above. β_1_AR-m23 was eluted from Ni^2+^-NTA agarose with 300 mM imidazole and all samples analysed by SDS-PAGE. Also in these experiments, the length of digestion, both on the column and in bulk solution, was increased to 3 min to compensate for the diminished activity of thermolysin in high concentrations of urea.

## Results

### Urea unfolding of β_1_AR-m23 in DM in bulk solution and on a solid support

Intrinsic protein fluorescence and far-UV circular dichroism (CD) were used to monitor changes in β_1_AR-m23 structure in bulk solution in DM and in the presence of the chemical denaturant urea. Unfolding was assessed by a reduction in helical secondary structure (as shown by a decrease in the 222 nm CD band characteristic of α helical structure). The change in tryptophan fluorescence was also monitored during denaturation, as an increase in exposure of tryptophan residues to water is accompanied by a red-shift in the fluorescence emission maximum to longer wavelengths. Although the latter fluorescence change cannot necessarily be linked directly to changes in folded and unfolded state populations.

**Fig 1:**
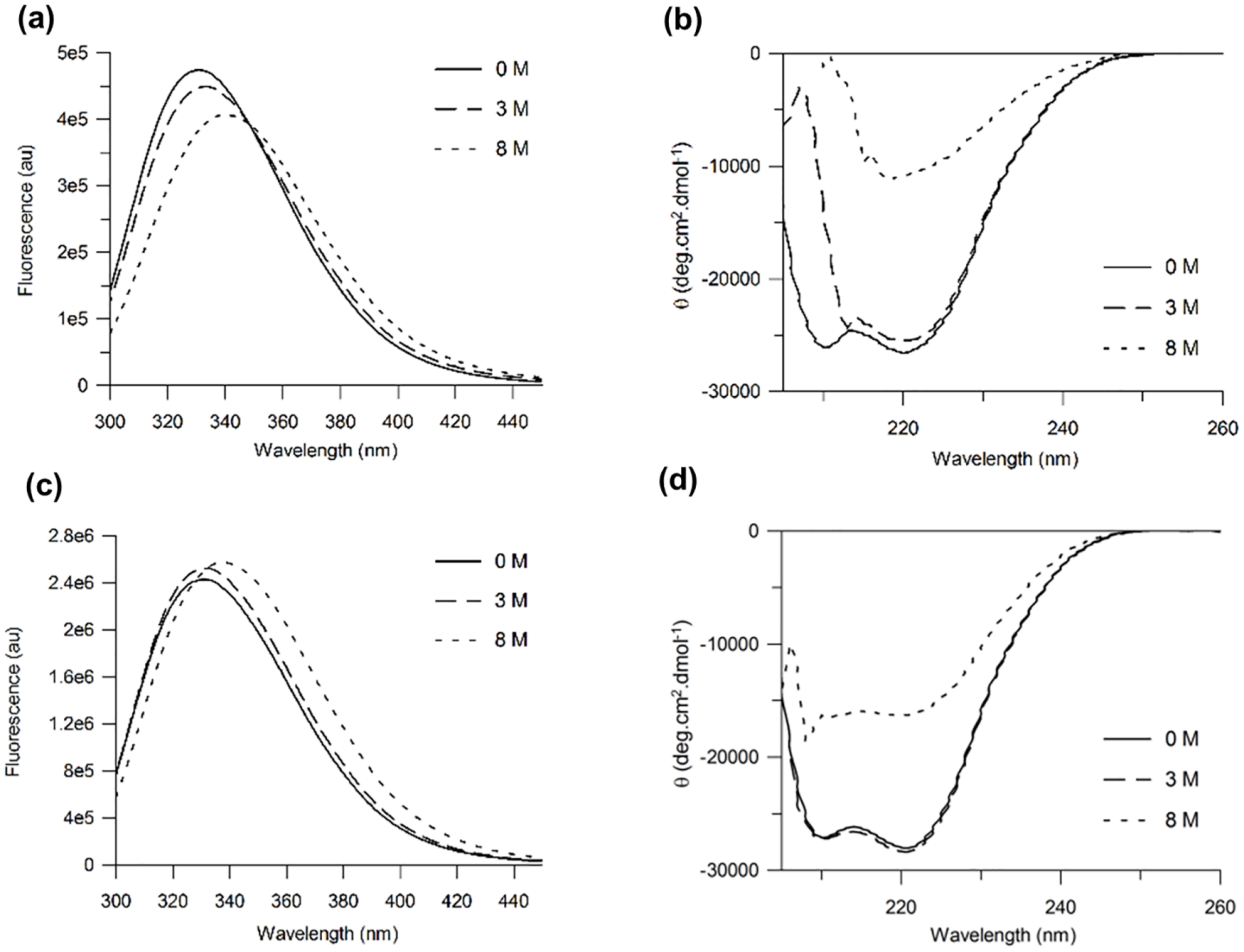
β_1_AR-m23 unfolding in DM and in urea. Fluorescence and far UV CD spectra (a and b, respectively) of 0.45 μM and (c and d, respectively) 4.5 μM β_1_AR-m23 in DM in the original folded state in the presence of 0 M urea (solid lines) and 3 M urea (dashed lines), and unfolded in 8 M urea (dotted lines). All buffers contained 25 mM Tris pH 7.5, 150 mM NaCl, 0.1 mM EDTA and 0.5% DM. Spectra are averages from a minimum of three independent experiments on different samples. For folded protein (in 0 M urea) the band intensity at 222 nm was -26070 ± 2400 deg.cm^2^.dmol^-1^ and -27780 ± 5400 deg.cm^2^.dmol^-1^ at a protein concentration of 0.45 μM and 4.5 μM, respectively. The wavelength at the fluorescence emission maximum for folded protein was 329.9 ± 0.8 nm and 329.8 ± 0.3 nm at a protein concentration of 0.45 μM and 4.5 μM, respectively. The intensity at the fluorescence emission maximum for folded protein was 474000 ± 118000 and 2404000 ± 399000 at a protein concentration of 0.45 μM and 4.5 μM, respectively.

Dilution of 0.45 μM β_1_AR-m23 in DM into buffer containing 8 M urea resulted in a protein fluorescence band that had red-shifted by ~ 9 nm (from 329.9 nm in DM to 339.3 nm in urea) (Fig 1a). Under the same conditions a decrease in intensity of the 222 nm CD band, from ~ -26,070 deg.cm^2^.dmol^-1^ to -10,480 deg.cm^2^.dmol^-1^, was observed corresponding to a loss of ~60% of its starting α-helix (Fig 1b). Plots of the red-shift in the fluorescence emission maximum correlated with those for reduction in helicity against urea concentration. These fluorescence and CD data exhibit similar midpoints of unfolding, *C*_*m*_, centred around 4.0 ± 0.1 M and 4.5 ± 0.1 M, respectively, and similar slopes at the *C*_*m*_ of 0.90 ± 0.07 nmM^-1^ and 0.82 ± 0.06% reduction in helicityM^-1^, respectively (S1a, S1b and S2 Figs). Unfolding was also carried out at a 10-fold higher protein concentration (4.5 μM) (Fig 1c and 1d). Under these conditions the receptor loses ~47% of its starting α-helix (Fig 1d).

**Fig 2:**
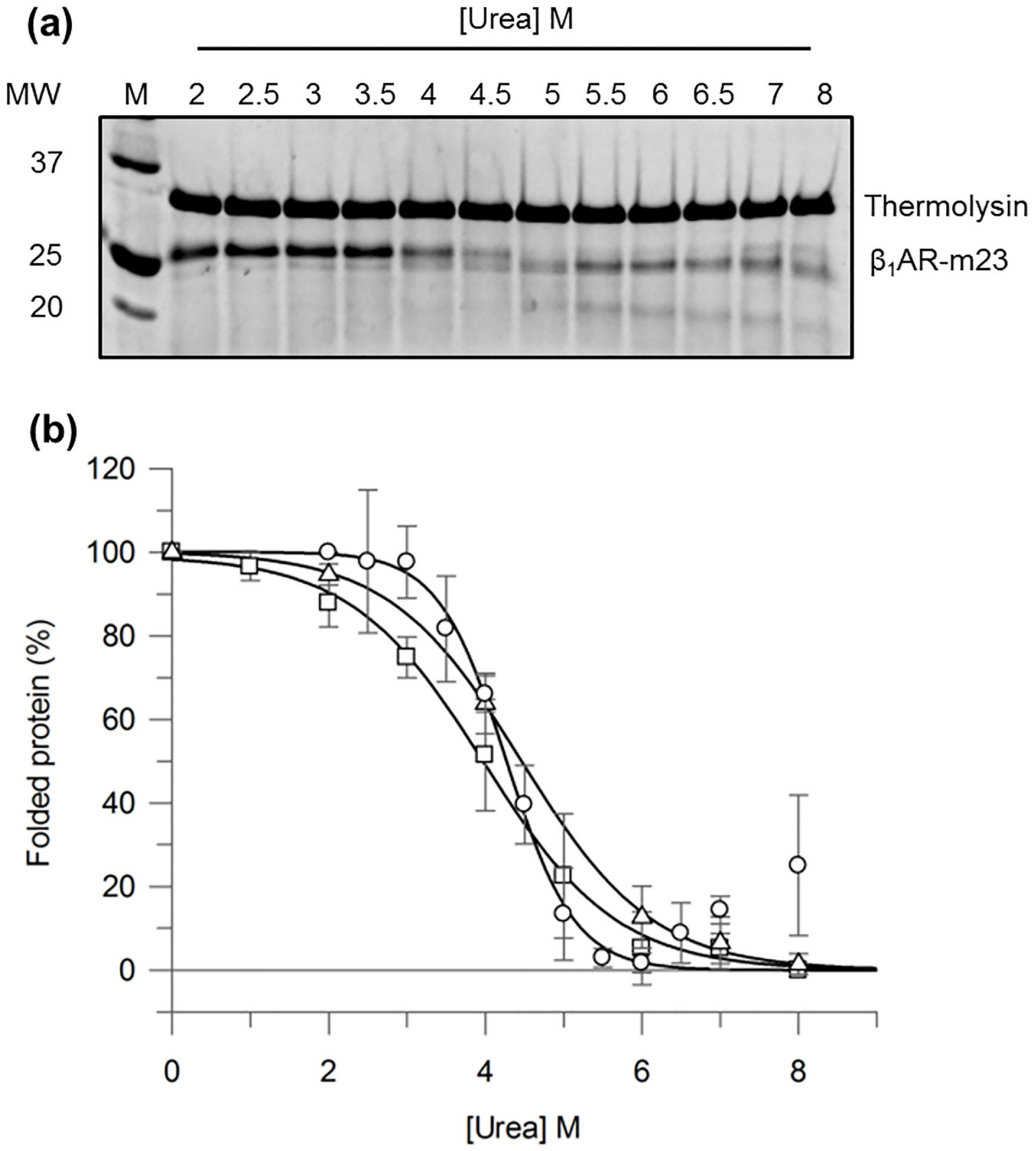
Monitoring β_1_AR-m23 unfolding in bulk solution by pulse proteolysis. 0.2 mg/ml thermolysin was used to digest 0.45 μM β_1_AR-m23, pre-equilibrated in buffer containing 25 mM Tris pH 7.5, 150 mM NaCl, 0.1 mM EDTA, 0.5% DM, 10 mM CaCl_2_ and urea (2–8 M) for 30 min, for 1 min. (a) A representative SDS-PAGE gel of β_1_AR-m23 following 1-min pulse proteolysis. (b) The percentage of folded β_1_AR-m23 remaining as determined by pulse proteolysis (circles), fluorescence (squares) and far-UV CD (triangles). The percentage of folded protein at each urea concentration was determined by; pulse proteolysis from the amount of undigested protein as measured by SDS-PAGE; fluorescence from the red-shift in the fluorescence emission maximum; and CD from the degree of α-helical structure as measured by the CD intensity at 222 nm. Resulting values were normalised between 0% and 100% with 100% representing the fully folded protein in DM and the 0% the partly unfolded 8 M urea state that possesses some helical content. Error bars show ± SD (standard deviation) and are the result of a minimum of three independent experiments on different samples.

Urea unfolding of β_1_AR-m23 in DM was also assessed on a solid support by binding to an affinity resin composed of Ni^2+^-NTA agarose beads by virtue of a His tag. As the beads caused light scattering artefacts in the spectroscopic measurements described above; pulse proteolysis was employed to monitor structural changes in the immobilised receptor in the presence of urea. Pulse proteolysis exploits differences in proteolytic susceptibility between folded and unfolded proteins [28, 29]. Following unfolding, proteolysis is performed briefly allowing digestion of unfolded protein while keeping folded protein intact. The fraction of folded protein can then be quantified by SDS-PAGE and plotted against denaturant concentration to determine *C*_*m*_. The validity of this method for monitoring β_1_AR-m23 unfolding in urea was initially tested in bulk solution using a 1 min pulse. The SDS-PAGE in Fig 2a shows the amount of folded receptor remaining in varying urea concentrations following 1-min pulse proteolysis with thermolysin. β_1_AR-m23 remains largely intact in 2–3 M urea (folding conditions) with band intensities comparable to that of undigested receptor. In higher urea concentrations (3–7 M) the fraction of folded receptor decreases, as demonstrated by the disappearance of the intact protein band and appearance of lower molecular weight cleavage products. In 7 M urea and above the intact protein band begins to reappear, probably due to the reduced activity of thermolysin in high urea [28, 30]. The *C*_*m*_ value obtained by pulse proteolysis (4.3 ± 0.1 M with an associated slope of 0.4 ± 0.1% folded proteinM^-1^) is in excellent agreement with that determined by fluorescence and far-UV CD (Fig 2b and Table 1). It should be noted, that in the complete absence of urea some folded β_1_AR-m23 was digested. Although this could be overcome by adding less protease this also reduced the sensitivity of the assay.

**Table 1:** *C*_*m*_ values for 0.45 μM β_1_AR-m23 unfolded in urea as determined by 1-min pulse proteolysis, fluorescence and circular dichroism.

	*C*_*m*_ [M]	Slope [% folded protein M^-1^]
Pulse proteolysis	4.3 ± 0.1	0.4 ± 0.1
Fluorescence	4.0 ± 0.4	0.9 ± 0.2
Circular dichroism	4.5 ± 0.3	0.8 ± 0.1

*C*_*m*_ values for 0.45 μM β_1_AR-m23 unfolded in urea as determined by 1-min pulse proteolysis, fluorescence and circular dichroism. Unfolding curves in Fig 2 were fit to determine the denaturant concentration at the midpoint of unfolding (*C*_*m*_) together with the slope at the *C*_*m*_. Errors are ± SD and are the result of a minimum of 3 independent experiments on different samples.

**Fig 3:**
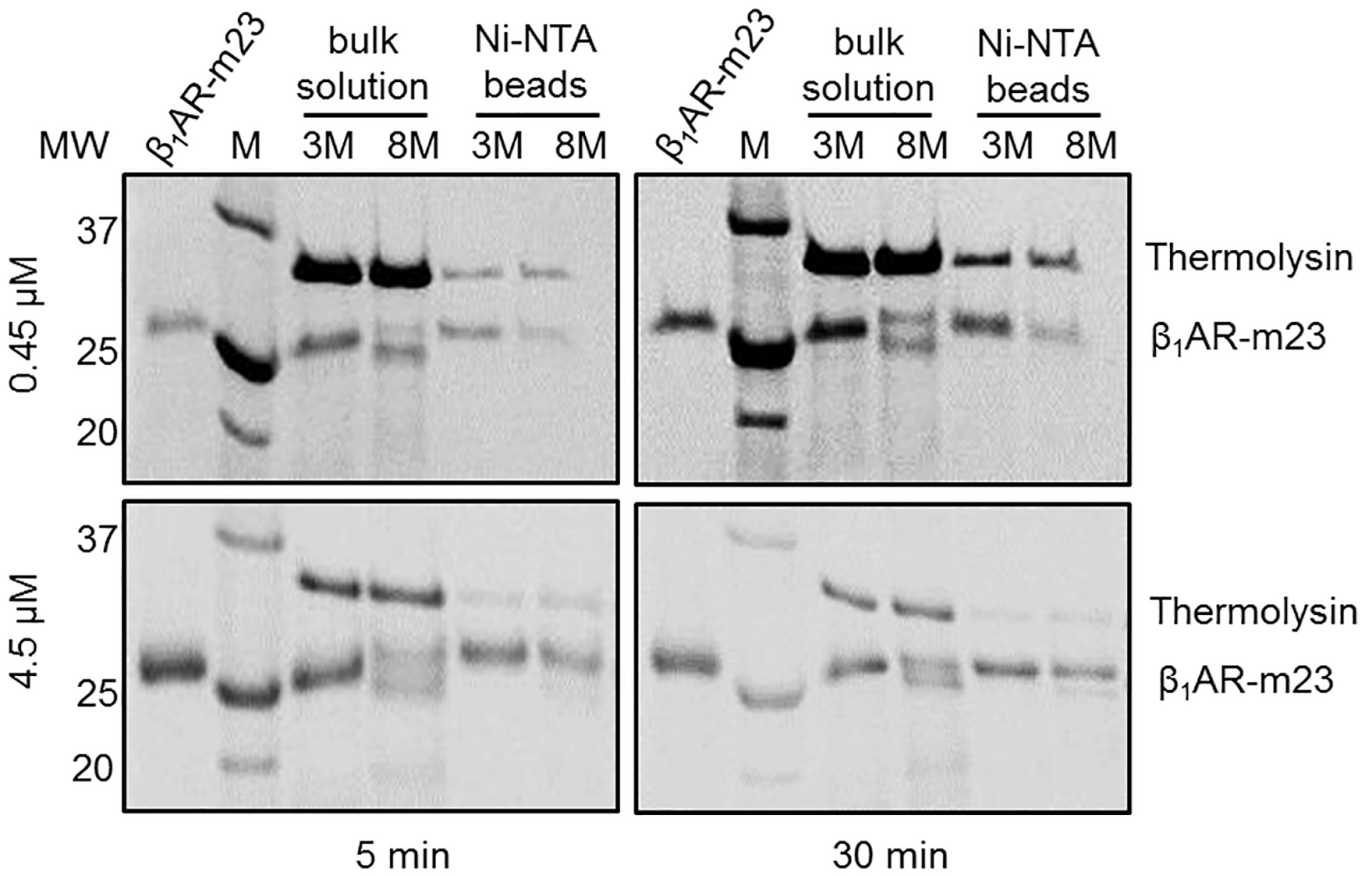
Comparing β_1_AR-m23 unfolding in bulk solution and on a solid support by pulse proteolysis. Example SDS-PAGE gels of β_1_AR-m23 following 3-min pulse proteolysis after a 5 or 30 min incubation in urea. Briefly, 0.45 or 4.5 μM β_1_AR-m23 in either bulk solution or bound to Ni^2+^-NTA column was equilibrated with buffer containing 25 mM Tris pH 7.5, 150 mM NaCl, 0.1 mM EDTA, 0.5% DM, 10 mM CaCl_2_ and 3 (folding conditions) or 8 M (unfolding conditions) urea followed by digestion with 0.2 mg/ml thermolysin for 3 min. The original folded β_1_AR-m23 is shown for comparison (*lanes β*_*1*_*AR-m23*) and molecular mass markers are indicated in kDa (*lanes M*).

Pulse proteolysis was also used to monitor β_1_AR-m23 unfolding on a Ni^2+^-NTA column and compared to that in bulk solution. The length of digestion was increased to 3 min to compensate for the diminished activity of the protease in 8 M urea (used for unfolding). Following equilibration of 4.5 μM β_1_AR-m23 on a solid support under folding (3 M urea) or unfolding conditions (8 M urea), as judged by bulk solution fluorescence and CD experiments (Fig 1c and 1d), clear differences in proteolytic susceptibility were observed; after a 5 min incubation in urea 60.3 ± 10.7% of the amount of receptor present in 3 M urea remained intact in 8M urea (Fig 3 and Table 2). The concentration of thermolysin used in these proteolysis experiments (~ 6 μM) was similar to that of the receptor meaning unfolded protein is likely to be digested slower than in conditions of an excess of protease. Experiments performed with higher concentrations of protease or with longer digestion periods resulted in greater problems with digestion of folded protein (data not shown). However, when the experiments were carried out with 10-fold less protein (0.45 μM), ensuring an excess of protease, greater difference in proteolytic susceptibility were observed in 3 and 8 M urea; only 41.5 ± 5.1% of the amount of receptor in 3 M urea remained intact in 8 M urea (Fig 3 and Table 2). β_1_AR-m23 unfolding on the column is further supported by its inability to bind [^3^H] (-) dihydroalprenolol (DHA) following incubation in 8 M urea (S4 Fig).

**Table 2:** The band intensities of intact β_1_AR-m23 on SDS-PAGE gels following 3-min pulse proteolysis in urea.

	0.45 μM β_1_AR-m23		4.5 μM β_1_AR-m23	
	5 min	30 min	5 min	30 min
Bulk solution	28.8 ± 7.5	31.3 ± 4.1	56.4 ± 11.8	42.1 ± 5.1
Ni-NTA beads	41.5 ± 5.1	40.2 ± 6.9	60.3 ± 10.7	59.8 ± 10.5

The band intensities of intact β_1_AR-m23 on SDS-PAGE gels following 3-min pulse proteolysis in 3 M (folding conditions) or 8 M (unfolded conditions) urea (Fig 3) were calculated using the AlphaErase FC software. Values given are the percentage amounts of intact β_1_AR-m23 remaining in 8 M (unfolded conditions) compared to in 3 M (folded conditions) urea. Each value is the result of an average of between 5 to 9 independent measurements on different samples; errors are ± SD.

### Reversible folding of β_1_AR-m23 in DM from a urea-denatured state

4.5 μM β_1_AR-m23 in 8 M urea, which has lost a significant amount (~47%) of its starting α-helical structure was used for refolding experiments (Fig 1d). Successful refolding was primarily assessed by recovery of ligand binding activity; to that of the purified receptor prior to unfolding. Recovery of the CD and fluorescence spectra to that of the original purified state was also used. Refolding was attempted into DM by two methods: (i) on a solid support using a Ni^2+^-NTA affinity column and (ii) in bulk solution by rapid dilution.

**Fig 4:**
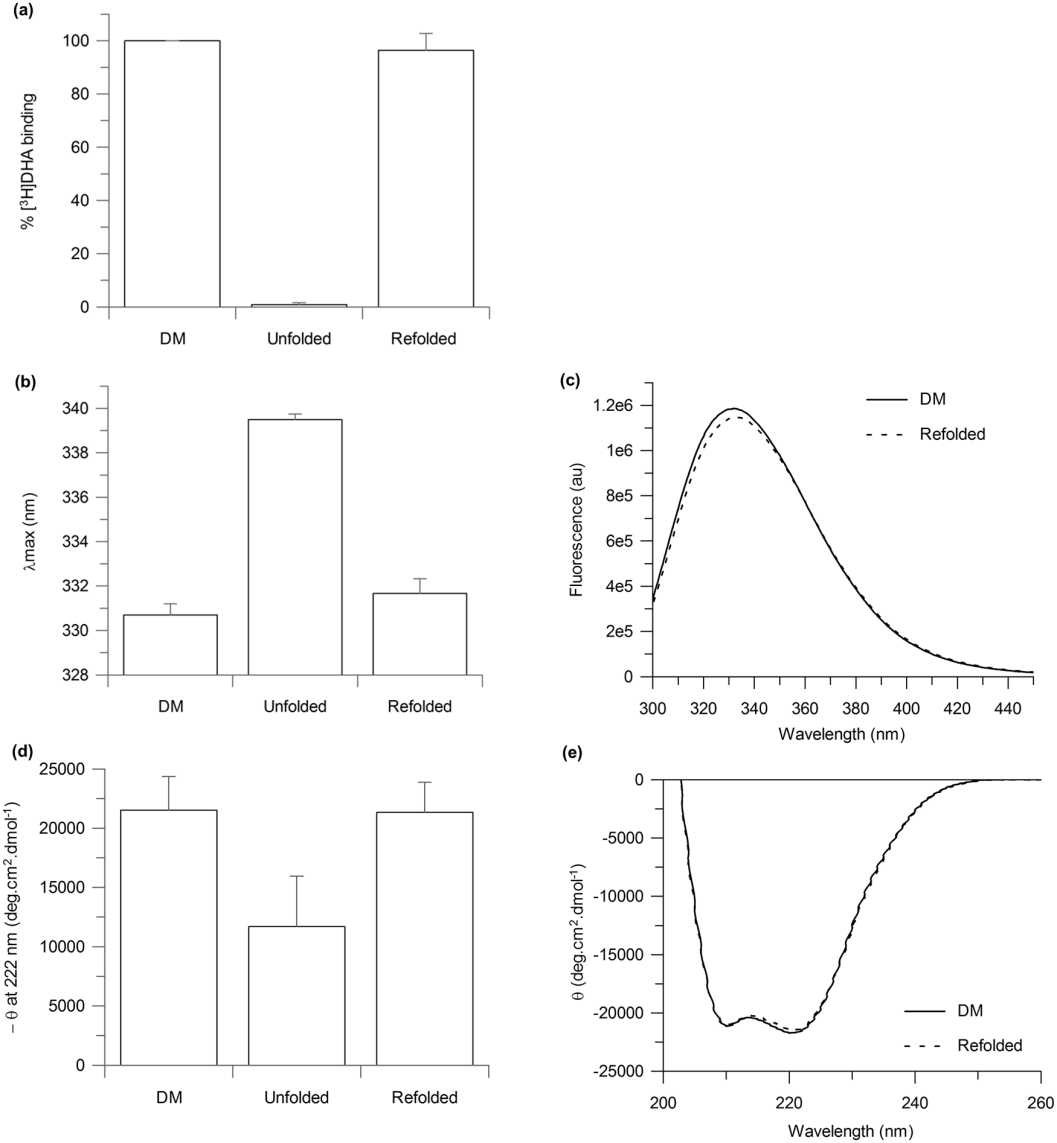
β_1_AR-m23 refolding on a Ni^2+^-NTA column into DM, from urea. 4.5 μM β_1_AR-m23, bound to a Ni^2+^-NTA column, was unfolded in 25 mM Tris pH 7.5, 150 mM NaCl, 0.5% DM and 8 M urea for 5 mins before refolding into buffer in which the urea had been omitted. The (a) binding of antangonist [^3^H]DHA to as well as the (b) fluorescence emission maximum and (d) CD signal at 222 nm of the refolded receptor. Results are compared to the originally folded β_1_AR-m23 in 0.5% DM and β_1_AR-m23 unfolded in 8 M urea. Error bars show ± SD and are the result of three independent experiments on different samples. (c) Fluorescence and (e) far UV-CD spectra of the original folded β_1_AR-m23 in 0.5% DM (solid line) and of refolded β_1_AR-m23 in 0.5% DM (dotted line). Spectra are the average of three independent measurements. The fluorescence emission maximum was 330.7 ± 0.5 nm for the original folded receptor and 331.7 ± 0.7 nm for the receptor refolded in DM. The CD signal at 222 nm was– 21520 ± 2840 deg.cm^2^.dmol^-1^ for the original folded receptor and– 21360 ± 2520 deg.cm^2^.dmol^-1^.

β_1_AR-m23 refolding on a solid support by removal of urea on a Ni^2+^-NTA affinity column was determined to be very efficient with recovery of approximately 100% of functional protein, as determined by the recovery of approximately 100% of the original binding activity, to the antagonist [^3^H]DHA, as compared to that of the originally folded receptor in DM prior to any treatment with urea (Fig 4a). As the predicted molecular weight of our receptor construct is 36 kDa, the theoretical maximum specific binding for pure receptor is 27.8 nmol/mg protein, assuming one binding site per receptor. Ligand binding values of 10.8 ± 1.9 nmol/mg were obtained for the original folded β_1_AR-m23 in DM, which corresponded to ~ 40% of the theoretical maximum. This is comparable to previously reported ligand binding values obtained for truncated β_1_AR (βAR 34-424/His6) of between 10–12 nmol/mg [27]. The ligand binding activity of the refolded β_1_AR-m23 was 10.5 ± 2.5 nmol/mg. Tryptophan fluorescence and far-UV CD spectra also returned to that of the originally folded receptor in DM (Fig 4b–4e).

Dilution of β_1_AR-m23 into various DM-containing buffers resulted in incomplete refolding as judged by the lack of recovery of the original fluorescence band; the fluorescence emission maximum after dilution of 335.2 nm was only slightly shifted from the urea-unfolded state at 337.8 nm, and did not fully recover to the 329.9 nm maximum of the originally folded receptor in DM (S5 Fig). This difference is too large to be attributed to the small amount (0.8 M) of urea remaining in these samples as demonstrated by unfolding experiments in which urea concentrations greater than 4 M are required to induce such changes in the fluorescence emission maximum or CD signal at 222 nm (S1a and S1b Fig).

## Discussion

An in depth knowledge of the structural and molecular basis of GPCR function is lacking, often owing to difficulties in their overexpression, purification and stabilisation. It is therefore crucial to develop new and efficient methodologies for obtaining large amounts of native-like, functional and stable GPCRs. Here, we address a fundamental facet of GPCR function, their folding, and report the functional folding (in terms of ligand binding and secondary structure) of the exemplary GPCR, β_1_AR-m23, from a chemically-denatured state. This has provided important insights into how to obtain and stabilise the correct fold of the receptor. Firstly, the most stabilising conditions for the isolated receptor, are not necessarily the best for folding; although bicelles containing the lipid DMPC were found to be the most stabilising conditions for β_1_AR-m23 (S2 and S3 Figs), no refolding occurred in any of the bicelle conditions tested but only in DM detergent micelles. Secondly, the extent of helical perturbations in the denatured state is not a good indication of the likelihood of successful refolding; β_1_AR-m23 cannot be fully refolded from an SDS-denatured state even though SDS induces fewer perturbations in helical structure than in urea. At 4.5 μM β_1_AR-m23 loses 34% of its starting α-helical structure in SDS compared to 47% in urea (S6d Fig) however, only ~ 36% of the original binding activity is restored upon refolding from this SDS denatured state on a Ni^2+^-NTA column (S7c Fig). We show that the complete refolding of β_1_AR-m23 is achieved into DM micelles, from a urea-denatured state, using an affinity column as a solid support. One possibility is that compared to refolding in bulk solution, a solid support is advantageous in preventing unwanted protein/protein interactions that favour aggregation. We cannot however, rule out other contributions for example electrostatic effects of the solid surface which may aid in protein folding. Practically, this work provides the groundwork for obtaining stable, functional GPCRS for further structural and functional characterisation.

Although there are only a limited number of examples of refolding studies with GPCRs, the use of columns in recovering functional GPCRs from inclusion bodies solubilised in harsh detergents such as SDS and urea has been reported previously [15–19]. However, no detailed structural information of the denaturant-solubilised state exists, nor has complete refolding and recovery of a native-like state been demonstrated; the leukotriene B_4_ (LTB_4_) receptor, BLT1, has been functionally folded into detergent-lipid mixed micelles composed of LDAO-asolectin with folding yields of ~ 20–30% (defined as the ratio of the amount of functional protein obtained after dissociation from the column to that initially immobilised, as determined by the affinity constant for LTB_4_ binding) [15, 18]; the serotonin receptor, 5-HT4a was folded into the functional conformation in DMPC/CHAPS bicelles with folding yields of ~25% (defined as the ratio of the amount of soluble protein obtained after dissociation from the column to that initially immobilised. Refolding was also assessed at a qualitative level by analysing it’s ligand-binding properties) [17]; the olfactory OR5 receptor was first folded into digitonin detergent micelles with folding yields of ~ 80%, as judged by fluorescence-monitored ligand binding assays, before insertion into lipid vesicles [16]. In all these cases the protein was recovered from insoluble inclusion bodies. A similar column-assisted folding approach has also been employed to refold several other membrane proteins from inclusion bodies including the chloroplast protein-import channel, Toc 75, and the light harvesting complex II (LHC2). In both cases, the solubilised inclusion bodies were applied to the Ni^2+^ column and refolding initiated by buffer exchange from chaotrope to mild detergent; Triton X-100 (TX) for Toc 75 and n-octyl-β-D-glucopyranoside (OG) for LHC2. In the case of LHC2, this was followed by pigment binding and trimerisation upon transfer to mixed lipid-detergent micelles composed of TX and L-phosphatidyl-D,L-glycerol dipalmitoyl (PG) [31]. Refolding yields of 8% and 3% are reported, for Toc 75 and LHC2, respectively. Neither of these proteins could be refolded by dilution of the unfolded protein into detergent micelles or lipids. Here, β_1_AR-m23 is refolded from a urea-denatured state with known perturbations in both secondary and tertiary structure as well as abolition of ligand binding. Noteworthy, is that our folding studies are carried out under non-reducing conditions and thus the starting material for refolding also contains the two native disulphide bonds between the correct cysteine residues (C114-C199, and C192 -C198). Previous work on rhodopsin has shown that misfolding was caused by the formation of a disulphide bond different from that in native rhodopsin [32]. This level of structural information is important if we are to gain a more detailed understanding of GPCR folding mechanisms. Moreover, we achieve complete, 100% recovery of ligand binding activity as compared to that of untreated receptor which has not been unfolded, which is vital given the tendency of GPCR stability and ligand binding to be disrupted when solubilised in detergents *in vitro*. To our knowledge, this is the first example of such a highly efficient and truly reversible folding system for a GPCR.

The efficiency of β_1_AR-m23 refolding on the beads from urea was found to be dependent on the initial unfolding time in urea; with higher refolding yields obtained following shorter unfolding times in urea (over the range of 5 to 60 min), as judged by the recovery of protein fluorescence and ligand binding activity (S8 Fig). 100% refolding yields with complete recovery of helical structure, protein fluorescence and ligand binding, as compared to the receptor in DM prior to any urea treatment, were only observed following 5 min unfolding in urea. Longer unfolding times of 10, 30 and 60 min caused the refolding yields to drop to 90, 72 and 30%, respectively. We used pulse proteolysis combined with electrophoresis to monitor structural changes in β_1_AR-m23 on the column after unfolding in urea for 5 and 30 min. Comparisons of protein band intensities in unfolding (8 M) and folding (3 M) urea concentrations showed the percentage amount of intact receptor remaining in unfolding conditions compared to folding conditions; 60.3 ± 10.7% and 59.8 ± 10.5% of the of the intact protein remained after 5 and 30 min unfolding, respectively (Fig 3 and Table 2), thus implying no further unfolding with a longer unfolding time than 5 min.

Interestingly, β_1_AR-m23 cannot be fully refolded from an SDS-denatured state. Previous reports have found recovery of functional GPCRs from SDS-solubilised inclusion bodies. Much lower yields have been reported for obtaining functional GPCRs from urea-solubilised inclusion bodies Work by Rogl *et al*. reporting on the refolding of Toc75 from bacterial inclusion bodies also suggests that inclusion bodies are more resistant to unfolding compared to their folded counterparts; tryptophan fluorescence spectroscopy was used to show that the unfolded inclusion bodies had an emission peak that was intermediate (346 nm) to that of folded Toc 75 (342 nm) and the same sample unfolded in urea (353 nm) [31]. Our more defined folding system seems to be the key to achieving higher refolding yields and may be applicable to other GPCRs, as well as for membrane proteins in general.

Although our understanding of the various factors affecting the folding yields of GPCRs is still somewhat limited, our results support the idea that preventing aggregation through the formation of intermolecular protein interactions, facilitates folding. This can be achieved by carrying out folding with the receptor immobilised on a solid support, for example on a Ni^2+^ affinity resin if the protein carries a His-tag and by limiting the length of time that the receptor is in denaturant. Given the large variation in GPCR folding environments reported, it may be that the choice of this environment is specific to each receptor in which case the most straightforward strategy is to systematically test a range of folding conditions and quantify the associated refolding yields, as demonstrated here.

## Supporting Information

S1 Appendix Supporting Methods and Results. (DOCX)

S1 Fig β_1_AR-m23 unfolding in DM-containing buffers. Fluorescence and far-UV CD were used to monitor unfolding in the presence of urea and SDS and various conditions screened in their ability to improve the resistance of the receptor against denaturation. Plots of the red-shift in the fluorescence emission maximum (λ_max_) *versus* concentration of (a) urea and (c) SDS and plots of the reduction in helicity *versus* concentration of (b) urea and (d) SDS are shown. The reduction in helicity is determined from the reduction in CD signal at 222 nm. Unfolding was carried out at a β_1_AR-m23 concentration of either 0.45 μM or 4.5 μM in buffers containing 25 mM Tris pH 7.5, 150 mM NaCl, 0.1 mM EDTA, 0.5% DM and varying concentrations of urea (0–9 M), unless stated otherwise, for 30 min. Plots show unfolding under the following conditions; at a final protein concentration of 0.45 μM (open circles, solid line) and at the same protein concentration but in the presence of 350 mM NaCl (open squares, dashed line), 10% (w/v) glycerol (open triangles, dotted line), 0.02% (w/v) CHS (closed circles, solid line) and 1 μM alprenolol (solid squares, dashed line) and at a final protein concentration of 4.5 μM (solid triangles, dotted line). Error bars show ± SD.
(TIF)

S2 Fig Fluorescence- and CD-measured *C*_*m*_ values for β_1_AR-m23 unfolding in urea and SDS. (TIF)

S3 Fig β_1_AR-m23 unfolding in DMPC/CHAPS. Plots of the red-shift in the fluorescence emission maximum (λ_max_) *versus* concentration of (a) urea and (b) SDS. Unfolding was performed at a final receptor concentration of 0.45 μM in bicelles of varying *q* values; 0.49 (open circles), 0.91 (open squares), 1.68 (open triangles), 2.73 (closed circles) and 3.63 (closed squares), or in DM (closed triangles). All buffers contained 2% (w/v total lipid and detergent) DMPC/CHAPS, 25 mM Tris pH 7.5, 150 mM NaCl and 0.1 mM EDTA. Error bars show ± SD.
(TIF)

S4 Fig Binding activity of β_1_AR-m23 on a Ni^2+^ -NTA column. The amount of radioligand bound to 4.5 μM β_1_AR-m23 on a Ni^2+^ -NTA column following 5 min incubation with buffer containing 25 mM Tris pH 8, 150 mM NaCl, 0.1 mM EDTA, 0.5% DM and 8 M urea (unfolded). Results are compared to control experiments carried out with no protein (no protein) and with β_1_AR-m23 but under folding conditions in the absence of urea (native). Error bar show ± SD and are the result of a minimum of four independent experiments on different samples.
(TIF)

S5 Fig Refolding β_1_AR-m23 by dilution into DM, from urea. Changes in the fluorescence emission maximum of β_1_AR-m23 initially unfolded at 4.5 μM in 8 M urea for 5 min and then diluted 10-fold into various DM-containing buffers all containing 25 mM Tris pH 7.5, 150 mM NaCl and 0.5% DM. Comparisons with that of the original folded β_1_AR-m23 in 0.5% DM and unfolded β_1_AR-m23 in 8 M urea are shown. Error bars show ± SD and are the result of three or four independent experiments on different samples.
(TIF)

S6 Fig β_1_AR-m23 unfolding in DM and in SDS. Fluorescence and far-UV CD spectra of (a and b, respectively) 0.45 μM and (c and d, respectively) 4.5 μM β_1_AR-m23 in 25 mM Tris pH 7.5, 150 mM NaCl, 0.1 mM EDTA and 0.2% DM in the original folded state in the absence of SDS (solid lines) and unfolded in 0.84 *X*_SDS_ (~0.65% SDS) (dotted lines). Fluorescence and CD spectra show the results from a single measurement. For folded protein (in 0 *X*_SDS_) the CD signal at 222 nm was -18990 deg.cm^2^.dmol^-1^ and– 21530 deg.cm^2^.dmol^-1^ at a protein concentration of 0.45 μM and 4.5 μM, respectively. The wavelength at the fluorescence emission was 330.4 nm and 328. 5 nm at a protein concentration of 0.45 μM and 4.5 μM, respectively. The intensity at the fluorescence emission maximum was 516000 and 3404000 at a protein concentration of 0.45 μM and 4.5 μM, respectively. Folding experiments with SDS were carried out using a different protein preparation to those carried out with urea and were not pursued in great depth due to more successful refolding results achieved with urea.
(TIF)

S7 Fig Refolding β_1_AR-m23 into DM, from SDS. Refolding of 4.5 μM β_1_AR-m23 was performed following a 5 min incubation with 25 mM Tris pH 7.5, 150 mM NaCl, 0.1 mM EDTA, 0.2% DM and 0.84 *X*_SDS_ (0.65% SDS) in bulk solution, by rapid dilution or on a Ni^2+^-NTA column. (a) Fluorescence emission maxima of β_1_AR-m23 diluted 10-fold into various DM-containing buffers. (b) Fluorescence emission maxima and (c) binding of antagonist [^3^H](-)DHA to β_1_AR-m23 refolded on a column. Comparisons with that of original folded β_1_AR-m23 in 0.2% DM and unfolded β_1_AR-m23 in 0.84 *X*_SDS_ are shown. Error bars show ± SD and are the result of two or three independent experiments on different samples.
(TIF)

S8 Fig β_1_AR-m23 refolded on a Ni^2+^-NTA column into DM, from urea, over time. Changes in the intrinsic protein fluorescence and ligand binding activity of β_1_AR-m23 were measured as follows: (a) The fluorescence emission maximum (b) and binding of antagonist [^3^H]DHA of β_1_AR-m23 refolded into 25 mM Tris pH 7.5, 150 mM NaCl, 0.5% DM after a 5, 10, 30 and 60 min incubation with 8 M urea at a protein concentration of 4.5 μM. Results are compared to original folded β_1_AR-m23 in 0.5% DM and unfolded β_1_AR-m23 denatured in 8 M urea. Error bars show ± SD and are the result of two or three independent experiments on different samples.
(TIF)

## Abbreviations

β_1_AR-m23: thermostabilised β_1_-adrenergic receptor
CD: circular dichroism
CHAPS: 3-[(3-Cholamidopropyl)dimethylammonio]-1-propanesulfonate
CHAPSO: 3-[(3-Cholamidopropyl)dimethylammonio]-2-hydroxy-1-propanesulfonate
CHS: cholesteryl hemisuccinate
CMC: critical micelle concentration
DHA: [^3^H] (-) dihydroalprenolol
DM: n-decyl-β-D-maltoside
DMPC: L-α-1,2-dimyristoylphosphatidylcholine
GPCRs: G-protein coupled receptors
LTB_4_: leukotriene B_4_ receptor
OG: n-octyl-β-D-glucopyranoside
PG: L-phosphatidyl-D,L-glycerol dipalmitoyl
SDS: sodium dodecylsulfate
SD: standard deviation
TX: Triton X-100
