# Supplementary material for: Complete Reversible Refolding of a G-Protein Coupled Receptor on a Solid Support
Source: PLoS One. 2016 Mar 16;11(3):e0151582. doi: 10.1371/journal.pone.0151582 (PMC4794186; doi:10.1371/journal.pone.0151582)
Supplement: S1 Appendix — (DOCX) [file pone.0151582.s001.docx]

**Supporting Methods and Results**

**Supporting Methods**

**Bicelle preparation**

2 % (w/v) DMPC and CHAPS solutions were prepared by suspending the powder in 25 mM Tris pH 7.5, 150 mM NaCl and 0.1 mM EDTA and mixing at room temperature for 1 h. Appropriate volumes of DMPC and CHAPS were then combined to give the required mole fraction or q value in a 2% (total lipid and detergent) solution. Mixtures were then vortexed briefly and left to stir at room temperature for 1 h or until samples became clear. All bicelle samples were used within 24 h.

**Unfolding**

SDS unfolding in DM was carried out at a final β_1_AR-m23 concentration of 0.45 μM (0.0162 mg/ml) and 4.5 µM (0.162 mg/ml) by diluting β_1_AR-m23 in buffer containing 25 mM Tris pH 7.5, 150 mM NaCl, 0.1 mM EDTA and 0.2 % DM. For fluorescence unfolding in SDS, β_1_AR-m23 was diluted into buffer and then titrated with aliquots of 20 % (w/v) SDS in the same buffer. Fluorescence spectra was recorded after incubation for 5 min. For CD unfolding experiments in SDS, β_1_AR-m23 was diluted into buffer containing varying concentrations of SDS (0-0.84 *X*_SDS_ (0-0.65 % SDS)) and CD spectra recorded after incubation for 30 min. SDS concentrations are given as as a mole fraction of the total deteregent (*X*_SDS_) rather than bulk concentrations and were calculated using the total detergent concentration. For those unfolding experiments in 2 % (w/v total lipid and detergent) DMPC/CHAPS, β_1_AR-m23 purified in 0.1 % DM was first diluted into DMPC/CHAPS to dilute the DM well below the critical micelle concentration (CMC) (usually at least a 20x dilution) and incubated at room temperature for 30 min.

**Refolding**

Refolding of urea-unfolded β_1_AR-m23 by rapid dilution into 2% (w/v) DMPC/CHAPS was essentially carried out as described previously for refolding into DM. Refolding of SDS-unfolded β_1_AR-m23 was attempted following unfolding in 0.84 *X*_SDS_ (0.65 % SDS) by dilution into buffer containing 0.2 % DM as above or on a Ni^2+^ resin by buffer exchange and removal of denaturants.

**Supporting Results**

**Irreversible folding conditions:**

**Irreversible folding of β_1_AR-m23 in DM from an SDS-denatured state**

The refolding of β_1_AR-m23 in DM was also investigated from an SDS-denatured state. SDS was found to induce fewer changes in β_1_AR-m23 structure compared to urea; dilution of 0.45 µM β_1_AR-m23 in DM into 0.84 *X*_SDS_ (~0.65 % SDS *cf.* cmc is ~ 0.22 % (8 mM)) resulted in a ~ 7 nm red-shift in the protein fluorescence band (337.0 nm in SDS) (S6a Fig) and 36 % loss of its starting α-helix (222 nm CD band ~ -12, 600 deg.cm^2^.dmol^-1^) (Fig S6b). The *C_m_* values obtained from fluorescence and CD were the same, 0.4 ± 0.0 *X*_SDS_ (S1c, S1d Fig and S2 Fig). Refolding experiments from an SDS-denatured state were performed using 4.5 µM β_1_AR-m23 in 0.84 *X*_SDS_ (0.65 % SDS) which has lost ~ 34 % of its starting α-helix and shows significant differences in native fluorescence spectra (S6c and S6d Fig). Under these conditions refolding was only partly reversible on a Ni^2+^-NTA column with ~ 36 % of the original binding activity restored (S7c Fig). No refolding was observed in bulk solution by dilution into various DM-containing buffers including the presence of cyclodextrin which has been reported to help strip away SDS molecules from the unfolded proteins, leaving them free to refold [1] (S7a Fig).

**Irreversible folding of β_1_AR-m23 in DMPC/CHAPS from urea- and SDS-denatured states**

To date, a number of different GPCR folding environments have been reported including bicelles composed of certain mixtures of long- and short-chain phospholipids. The folding of β_1_AR-m23 was therefore screened in various bicelle systems and the results compared to that in DM. The effects of urea and SDS on β_1_AR-m23 in bulk solution in DMPC/CHAPS bicelles of varying sizes was first investigated before attempting to use bicelles for refolding purposes. The ratio of DMPC to CHAPS, known as the q value [2, 3], was increased (or decreased) to increase (or decrease) the radius of the DMPC bilayer fragment. In the presence of either urea or SDS, the resistance of the receptor to denaturation increased in bicelles with smaller q values and thus decreasing mole fractions of DMPC, as demonstrated by unfolding curves with higher *C_m_* values (S2 Fig and S3 Fig). Similar findings were also observed when the CHAPS in the bicelle was replaced by either CHAPSO or DHPC (S2 Fig). In all three bicelle systems tested (DMPC/CHAPS, DMPC/CHAPSO and DMPC/DHPC), the receptor showed greater resistance to denaturation, in either urea or SDS, compared to in DM (S2 Fig). Greatest resistance was observed in DMPC/CHAPS bicelles (q = 0.91 and 0.49 for urea and SDS, respectively) with *C_m_* values approximately 2-fold higher than in DM (at the same receptor concentration) (S2 Fig and S3 Fig). Bicelles with smaller *q* values have less DMPC and more CHAPS, CHAPSO or DHPC. Conversely, bicelles with larger *q* values resemble more a pure DMPC bilayer. It should be noted that CHAPS, CHAPSO and DHPC micelles alone are found destabilising to β_1_AR-m23, resulting in fluorescence spectra more similar to that of unfolded protein (data not shown). Instead greatest stability is imparted when there is delicate balance between the ratio of long- and short-chain phospholipid; a small amount of DMPC bilayer to protect the receptor from the destabilising effects of CHAPS, CHAPSO and DHPC alone, and conversely amounts of CHAPS, CHAPSO and DHPC which offer additional stability over a pure DMPC bilayer. We can rule out the likelihood of protein aggregation at higher q values as in all our experiments we have an excess of bicelles over protein, in addition no aggregation is observed. Despite the increased resistance to chemical denaturation in DMPC/CHAPS bicelles over DM, any attempts to refold the receptor in bulk solution, from urea or SDS, into bicelles were unsuccessful.

**References**

1. Otzen DE, Oliveberg M. A simple way to measure protein refolding rates in water. Journal of molecular biology. 2001;313(3):479-83. Epub 2001/10/26. doi: 10.1006/jmbi.2001.5039. PubMed PMID: 11676533.

2. McKibbin C, Farmer NA, Jeans C, Reeves PJ, Khorana HG, Wallace BA, et al. Opsin stability and folding: modulation by phospholipid bicelles. Journal of molecular biology. 2007;374(5):1319-32. Epub 2007/11/13. doi: 10.1016/j.jmb.2007.10.018. PubMed PMID: 17996895.

3. Vold RR, Prosser RS, Deese AJ. Isotropic solutions of phospholipid bicelles: a new membrane mimetic for high-resolution NMR studies of polypeptides. Journal of biomolecular NMR. 1997;9(3):329-35. Epub 1997/04/01. PubMed PMID: 9229505.
