# Supplementary figures and images for: Complete Reversible Refolding of a G-Protein Coupled Receptor on a Solid Support
Source: PLoS One. 2016 Mar 16;11(3):e0151582. doi: 10.1371/journal.pone.0151582 (PMC4794186; doi:10.1371/journal.pone.0151582)

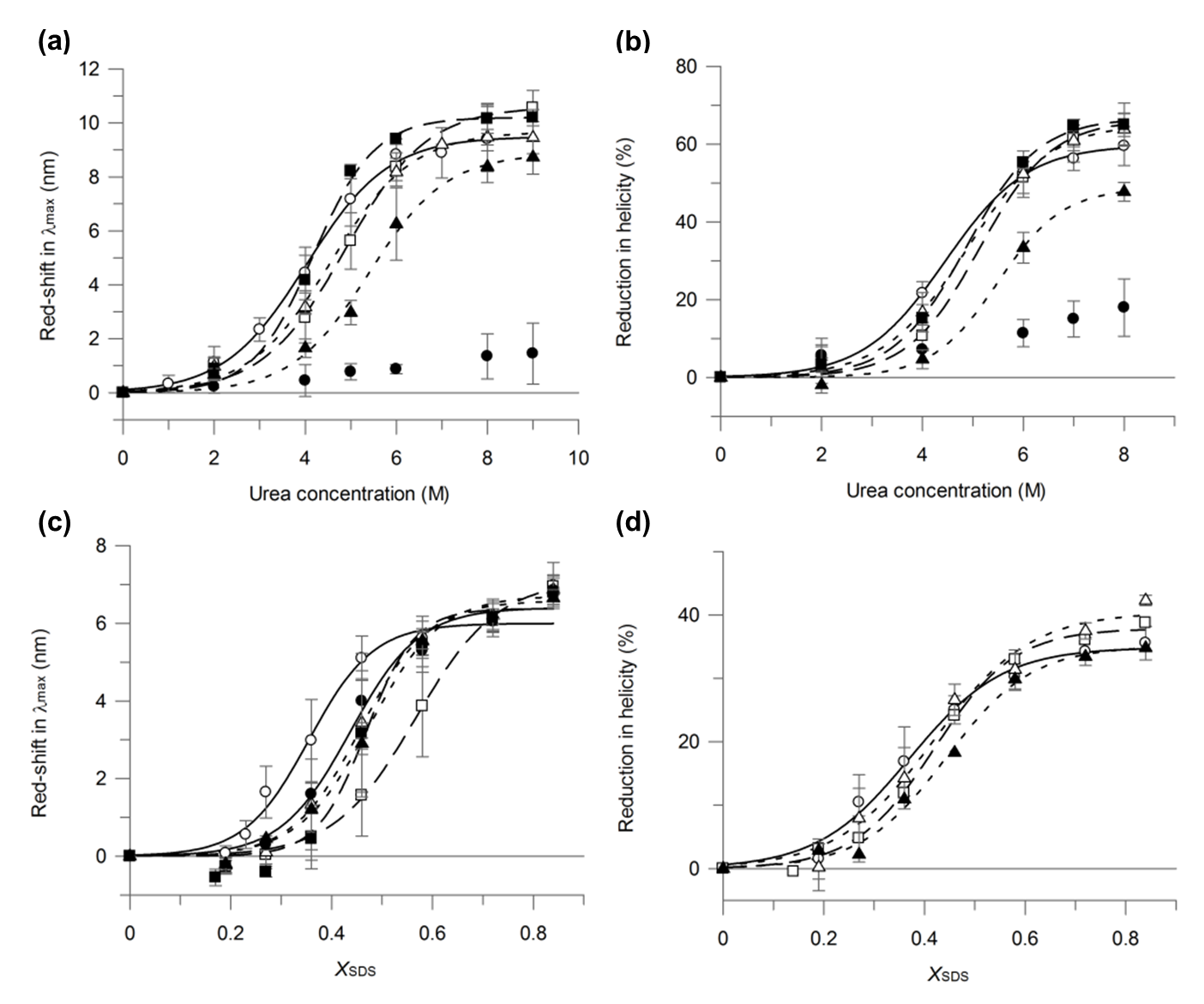

Supplement: S1 Fig — Fluorescence and far-UV CD were used to monitor unfolding in the presence of urea and SDS and various conditions screened in their ability to improve the resistance of the receptor against denaturation. Plots of the red-shift in the fluorescence emission maximum (λmax) versus concentration of (a) urea and (c) SDS and plots of the reduction in helicity versus concentration of (b) urea and (d) SDS are shown. The reduction in helicity is determined from the reduction in CD signal at 222 nm. Unfolding was carried out at a β1AR-m23 concentration of either 0.45 μM or 4.5 μM in buffers containing 25 mM Tris pH 7.5, 150 mM NaCl, 0.1 mM EDTA, 0.5% DM and varying concentrations of urea (0–9 M), unless stated otherwise, for 30 min. Plots show unfolding under the following conditions; at a final protein concentration of 0.45 μM (open circles, solid line) and at the same protein concentration but in the presence of 350 mM NaCl (open squares, dashed line), 10% (w/v) glycerol (open triangles, dotted line), 0.02% (w/v) CHS (closed circles, solid line) and 1 μM alprenolol (solid squares, dashed line) and at a final protein concentration of 4.5 μM (solid triangles, dotted line). Error bars show ± SD. (TIF) [file pone.0151582.s002.tif]

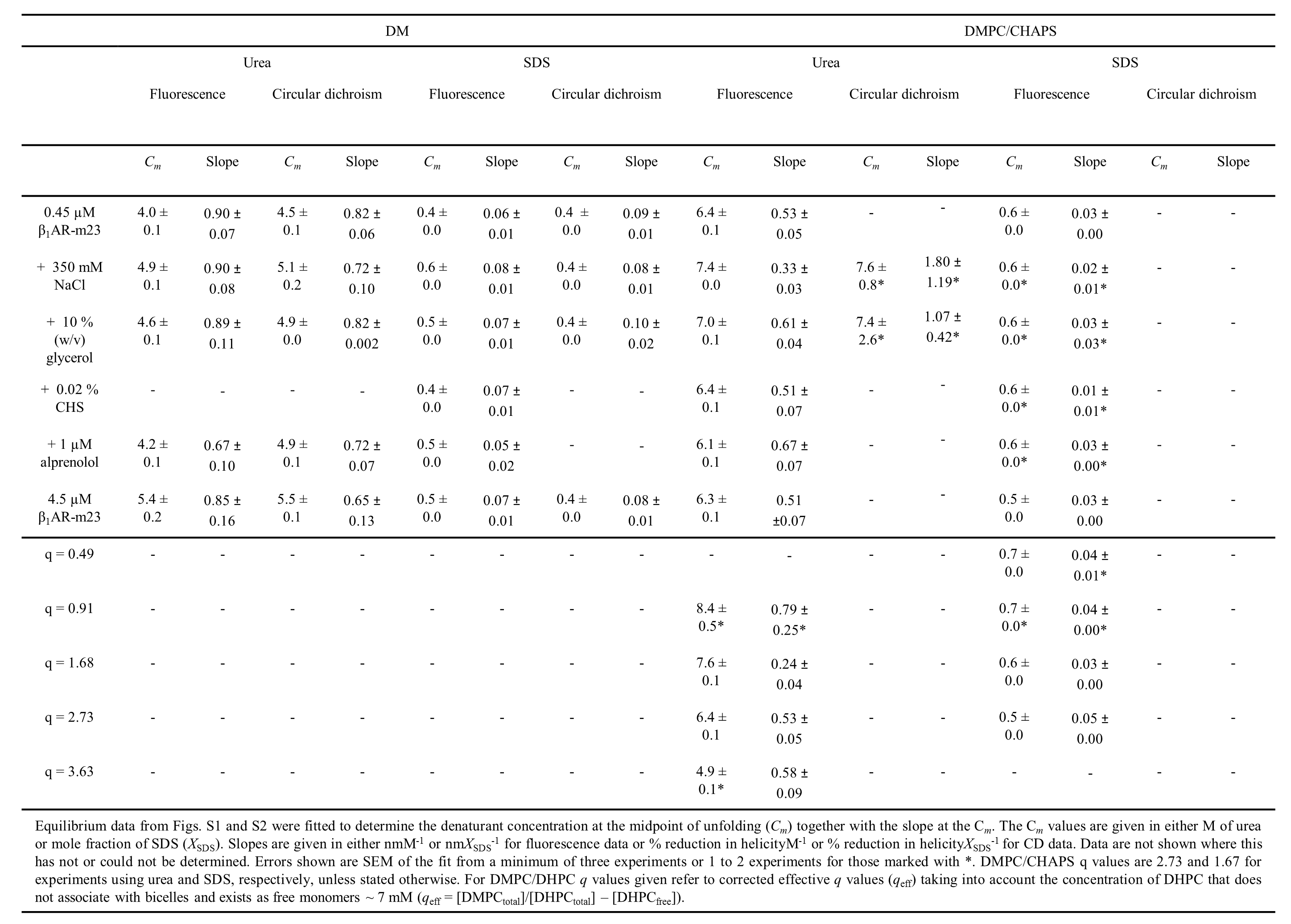

Supplement: S2 Fig — (TIF) [file pone.0151582.s003.tif]

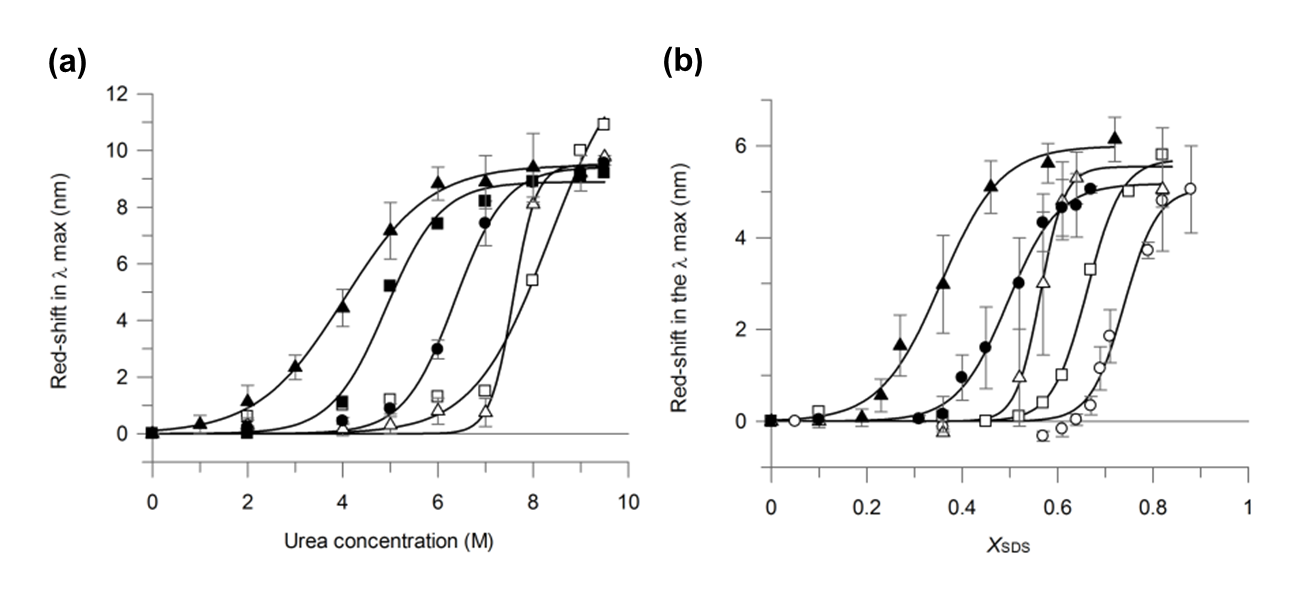

Supplement: S3 Fig — Plots of the red-shift in the fluorescence emission maximum (λmax) versus concentration of (a) urea and (b) SDS. Unfolding was performed at a final receptor concentration of 0.45 μM in bicelles of varying q values; 0.49 (open circles), 0.91 (open squares), 1.68 (open triangles), 2.73 (closed circles) and 3.63 (closed squares), or in DM (closed triangles). All buffers contained 2% (w/v total lipid and detergent) DMPC/CHAPS, 25 mM Tris pH 7.5, 150 mM NaCl and 0.1 mM EDTA. Error bars show ± SD. (TIF) [file pone.0151582.s004.tif]

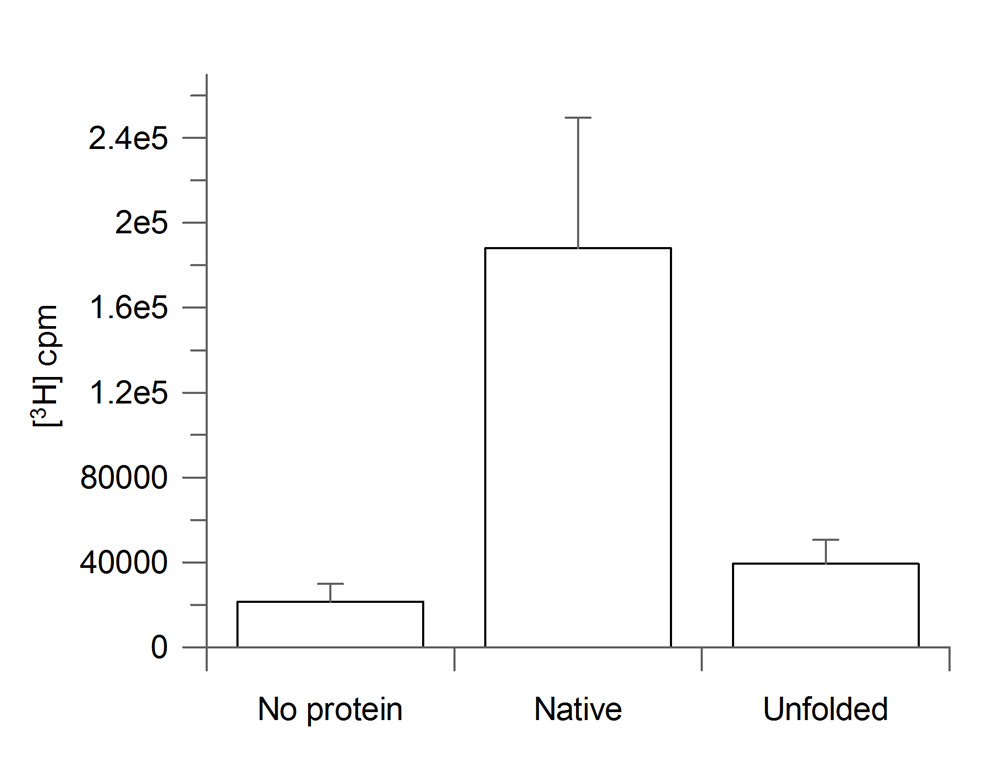

Supplement: S4 Fig — The amount of radioligand bound to 4.5 μM β1AR-m23 on a Ni2+ -NTA column following 5 min incubation with buffer containing 25 mM Tris pH 8, 150 mM NaCl, 0.1 mM EDTA, 0.5% DM and 8 M urea (unfolded). Results are compared to control experiments carried out with no protein (no protein) and with β1AR-m23 but under folding conditions in the absence of urea (native). Error bar show ± SD and are the result of a minimum of four independent experiments on different samples. (TIF) [file pone.0151582.s005.tif]

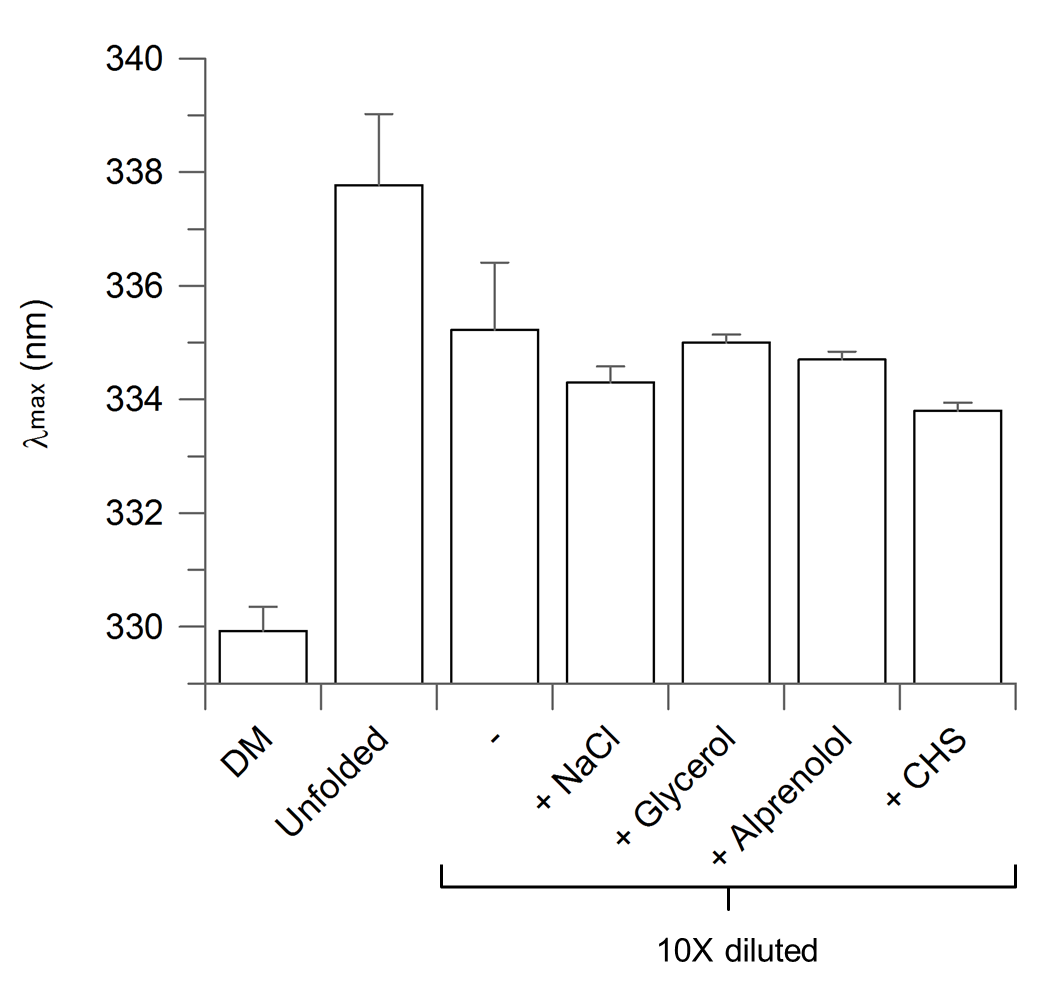

Supplement: S5 Fig — Changes in the fluorescence emission maximum of β1AR-m23 initially unfolded at 4.5 μM in 8 M urea for 5 min and then diluted 10-fold into various DM-containing buffers all containing 25 mM Tris pH 7.5, 150 mM NaCl and 0.5% DM. Comparisons with that of the original folded β1AR-m23 in 0.5% DM and unfolded β1AR-m23 in 8 M urea are shown. Error bars show ± SD and are the result of three or four independent experiments on different samples. (TIF) [file pone.0151582.s006.tif]

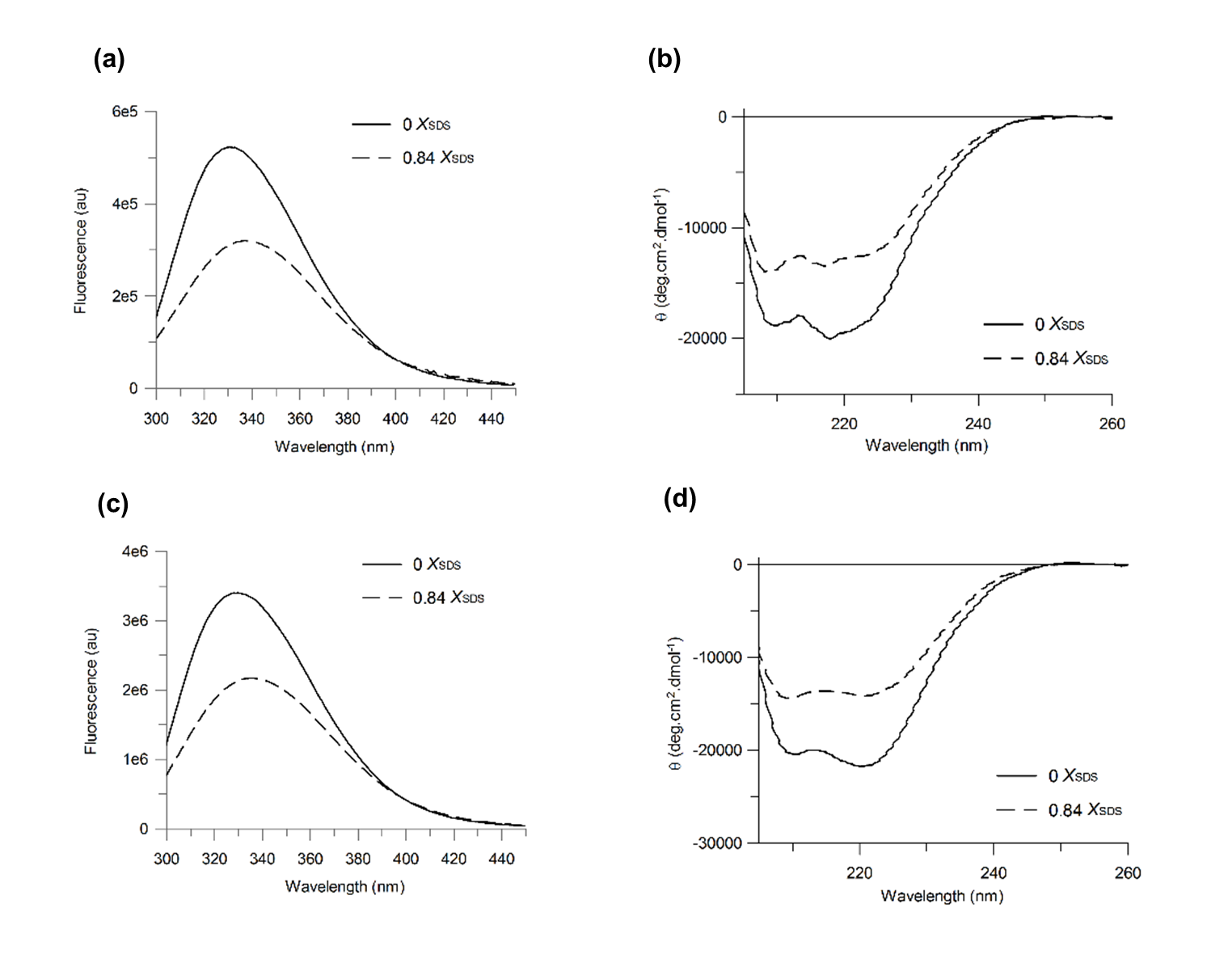

Supplement: S6 Fig — Fluorescence and far-UV CD spectra of (a and b, respectively) 0.45 μM and (c and d, respectively) 4.5 μM β1AR-m23 in 25 mM Tris pH 7.5, 150 mM NaCl, 0.1 mM EDTA and 0.2% DM in the original folded state in the absence of SDS (solid lines) and unfolded in 0.84 XSDS (~0.65% SDS) (dotted lines). Fluorescence and CD spectra show the results from a single measurement. For folded protein (in 0 XSDS) the CD signal at 222 nm was -18990 deg.cm2.dmol-1 and– 21530 deg.cm2.dmol-1 at a protein concentration of 0.45 μM and 4.5 μM, respectively. The wavelength at the fluorescence emission was 330.4 nm and 328. 5 nm at a protein concentration of 0.45 μM and 4.5 μM, respectively. The intensity at the fluorescence emission maximum was 516000 and 3404000 at a protein concentration of 0.45 μM and 4.5 μM, respectively. Folding experiments with SDS were carried out using a different protein preparation to those carried out with urea and were not pursued in great depth due to more successful refolding results achieved with urea. (TIF) [file pone.0151582.s007.tif]

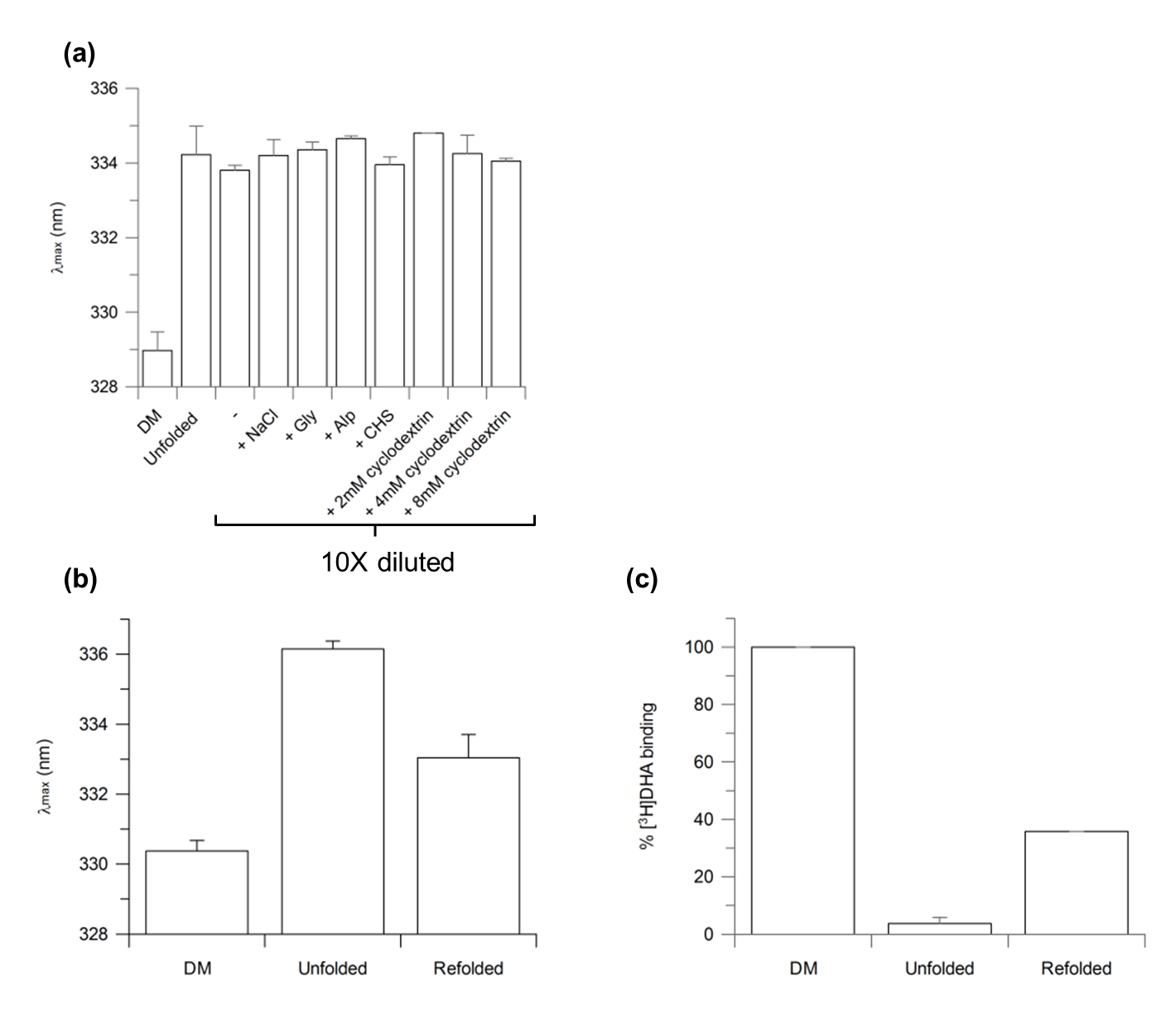

Supplement: S7 Fig — Refolding of 4.5 μM β1AR-m23 was performed following a 5 min incubation with 25 mM Tris pH 7.5, 150 mM NaCl, 0.1 mM EDTA, 0.2% DM and 0.84 XSDS (0.65% SDS) in bulk solution, by rapid dilution or on a Ni2+-NTA column. (a) Fluorescence emission maxima of β1AR-m23 diluted 10-fold into various DM-containing buffers. (b) Fluorescence emission maxima and (c) binding of antagonist [3H](-)DHA to β1AR-m23 refolded on a column. Comparisons with that of original folded β1AR-m23 in 0.2% DM and unfolded β1AR-m23 in 0.84 XSDS are shown. Error bars show ± SD and are the result of two or three independent experiments on different samples. (TIF) [file pone.0151582.s008.tif]

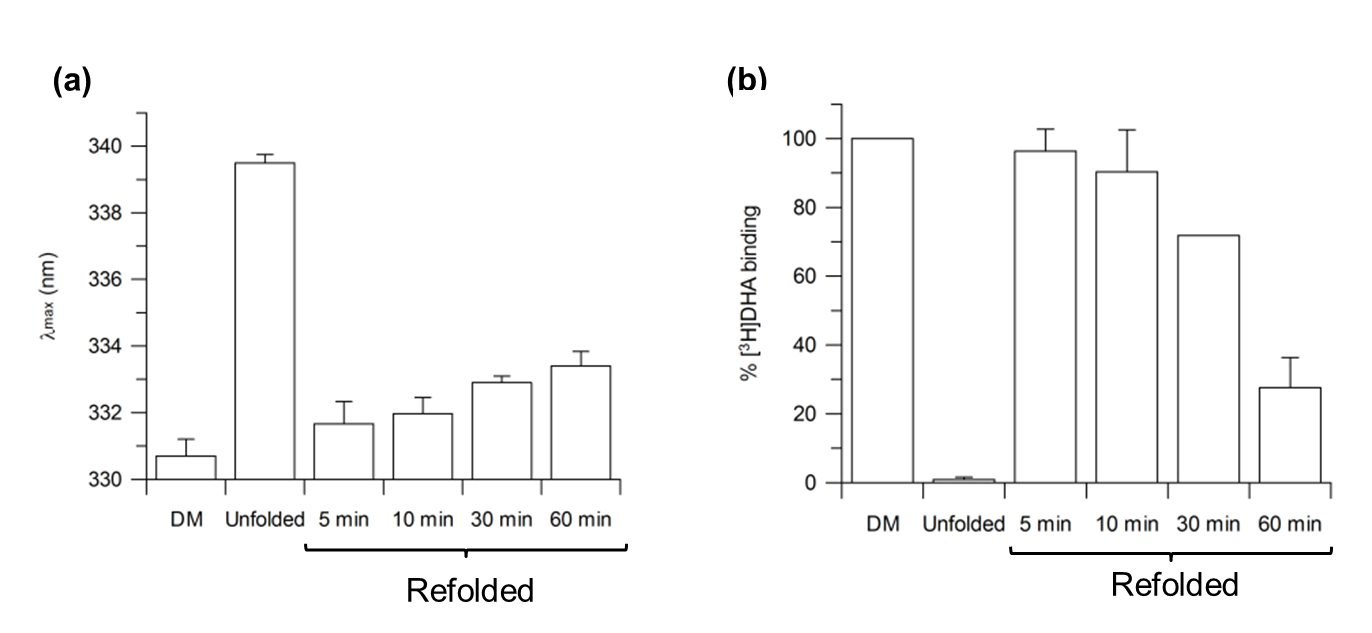

Supplement: S8 Fig — Changes in the intrinsic protein fluorescence and ligand binding activity of β1AR-m23 were measured as follows: (a) The fluorescence emission maximum (b) and binding of antagonist [3H]DHA of β1AR-m23 refolded into 25 mM Tris pH 7.5, 150 mM NaCl, 0.5% DM after a 5, 10, 30 and 60 min incubation with 8 M urea at a protein concentration of 4.5 μM. Results are compared to original folded β1AR-m23 in 0.5% DM and unfolded β1AR-m23 denatured in 8 M urea. Error bars show ± SD and are the result of two or three independent experiments on different samples. (TIF) [file pone.0151582.s009.tif]
